# Supplementary material for: Cheminformatics Identification of Phenolics as Modulators of Penicillin-Binding Protein 2a of Staphylococcus aureus: A Structure–Activity-Relationship-Based Study
Source: Pharmaceutics. 2022 Aug 29;14(9):1818. doi: 10.3390/pharmaceutics14091818 (PMC9503099; doi:10.3390/pharmaceutics14091818)
Supplement: Supplementary file 1 [file pharmaceutics-14-01818-s001.zip › pharmaceutics-1862418-supplementary.pdf]

Table S1: Consensus phenolic pharmacophore spatial arrangements

Pharmacophore class	x	y	z	Radius
Hydrogen donor	1.11	2.35	3.27	0.50
Hydrogen acceptor	2.82	1.43	1.10	0.50
Aromatic	5.87	-0.18	-0.23	0.10
Hydrophobic	0.11	0.12	0.98	1.00
Hydrophobic	5.87	-0.18	-0.23	1.00

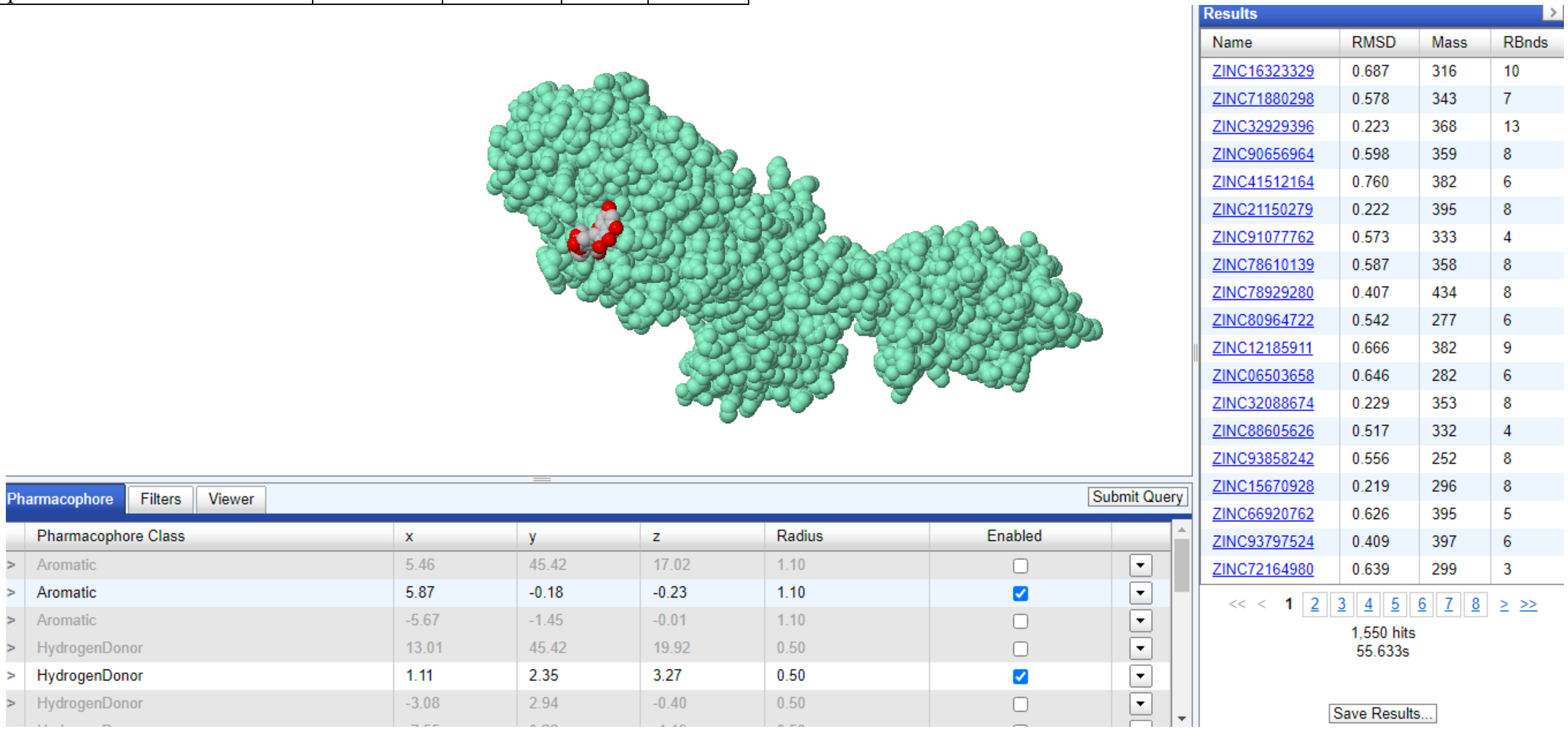

Figure S1: Observed 1550 hit compounds

Table S2: Average RMSD, ROG, and SASA of top five phenolics and amoxicillin following 120 ns simulation at the allosteric site of PBP2a of *S. aureus*

Systems	RMSD (Å)	RMSF (Å)	ROG (Å)	SASA (Å)
Unbound PBP2a	6.86 ± 1.18	2.71 ± 1.17	35.37 ± 0.45	26786.05 ± 473.91
PBP2a + Amoxicillin	3.34 ± 0.90	2.56 ± 1.32	37.16 ± 0.27	25452.79 ± 2301.39
PBP2a + Silicristin	3.48 ± 0.80	2.45 ± 1.11	36.93 ± 0.29	25466.88 ± 478.49
PBP2a + propan-2-one	4.17 ± 1.06	2.49 ± 1.14	36.12 ± 0.49	24802.25 ± 453.11
PBP2a + Epigallocatechin 4-benzylthioether	6.45 ± 2.33	3.48 ± 1.84	35.30 ± 0.88	25165.36 ± 509.79
PBP2a + chroman-4-one	3.24 ± 1.17	2.55 ± 1.32	36.89 ± 0.34	25844.78 ± 455.36
PBP2a + Epicatechin gallate	3.69 ± 0.68	2.06 ± 0.95	36.86 ± 0.21	25231.59 ± 459.17

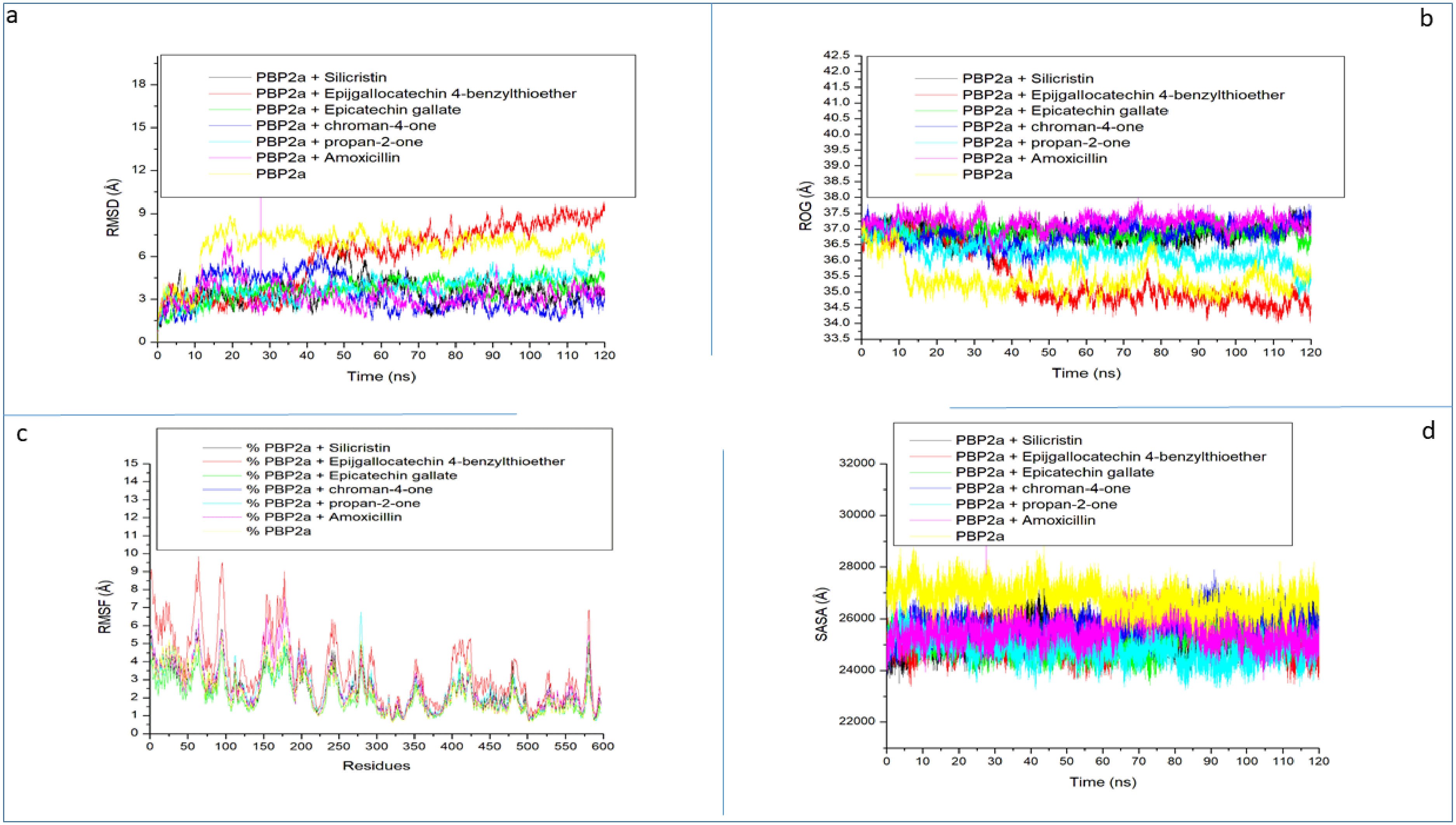

Figure S2: Comparative (a) root mean squared deviation (RMSD), (b) radius of gyration (ROG), (c) root mean squared fluctuation (RMSF), and (d) solvent-accessible surface area (SASA) plots of alpha-carbon, top five phenolics, and amoxicillin against the allosteric site PBP2a of *Staphylococcus aureus* over a 120 ns MD simulation period

Table S3: 2D plot interactions of the top five phenolics at the active and allosteric sites after 120 ns simulation

Ligands	2D plot interactions at the active site after 120 ns simulation	2D plot interactions at the allosteric site after 120 ns simulation
Amoxicillin	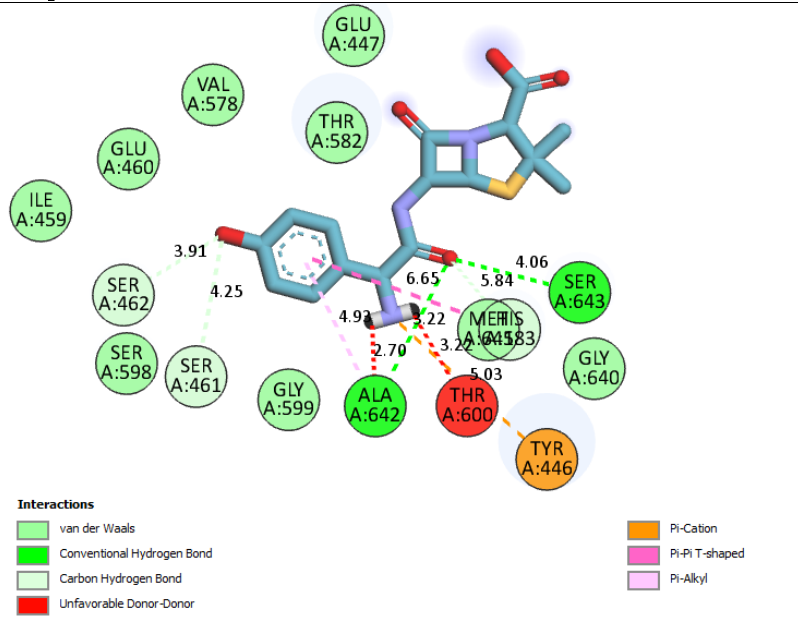	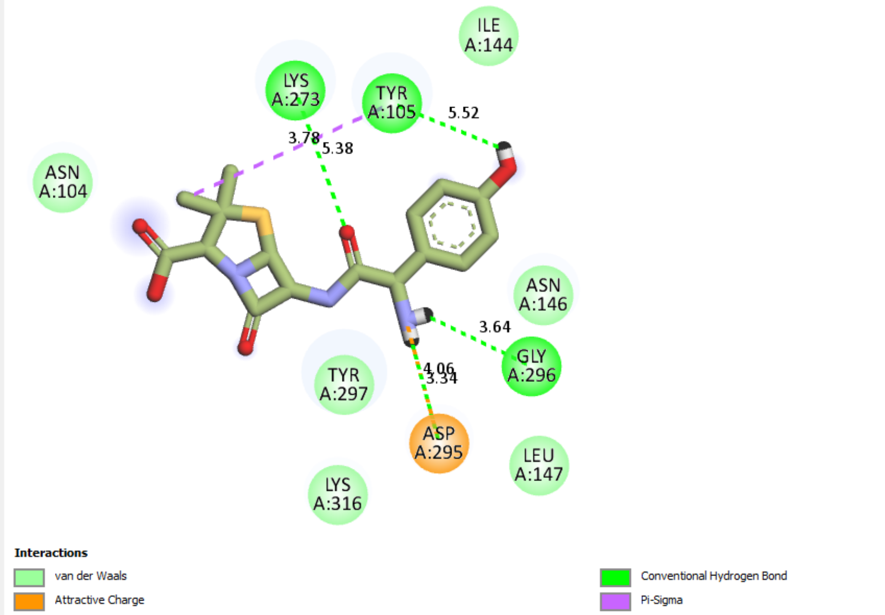

<table>
<tr>
<td>Silicristin</td>
<td>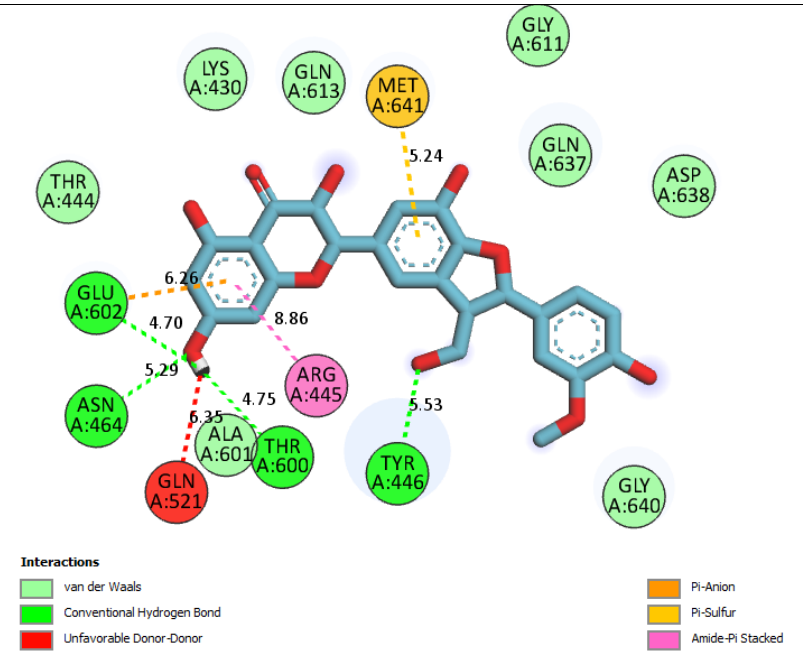
</td>
<td>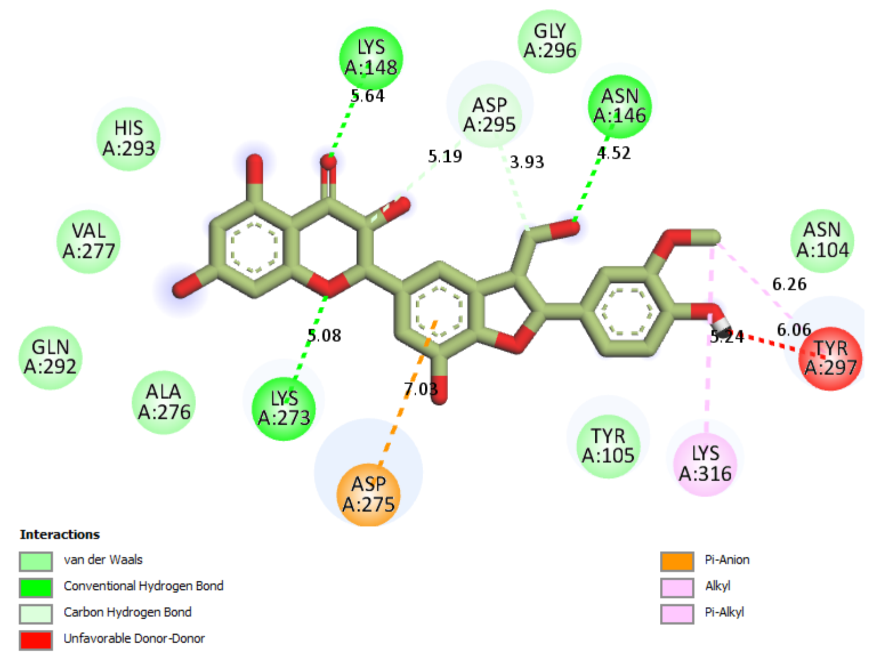
</td>
</tr>
</table>

Propan-2-one

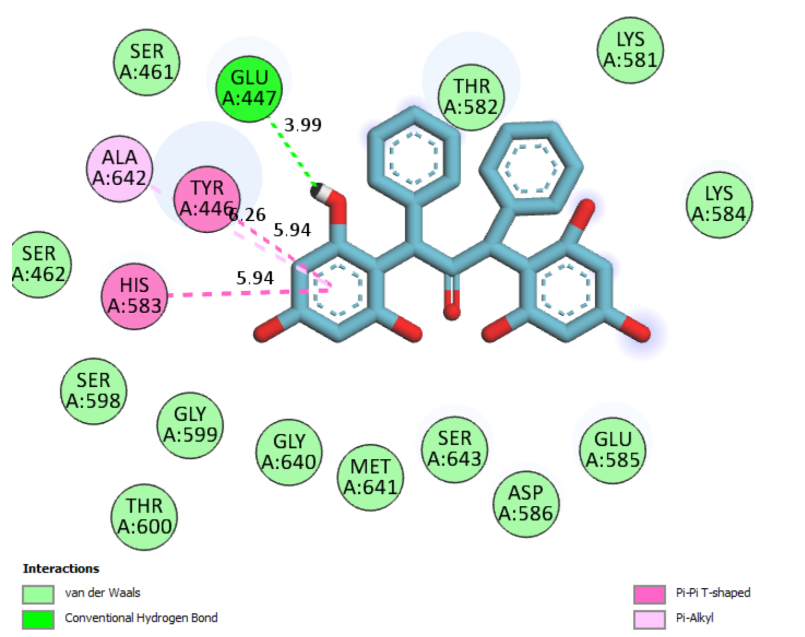
SER A:461
GLU A:447
THR A:582
LYS A:581
ALA A:642
TYR A:446
LYS A:584
SER A:462
HIS A:583
SER A:598
GLY A:599
GLY A:640
MET A:641
SER A:643
GLU A:585
ASP A:586
THR A:600
3.99
6.26
5.94
5.94
Interactions
van der Waals
Conventional Hydrogen Bond
Pi-Pi T-shaped
Pi-Alkyl

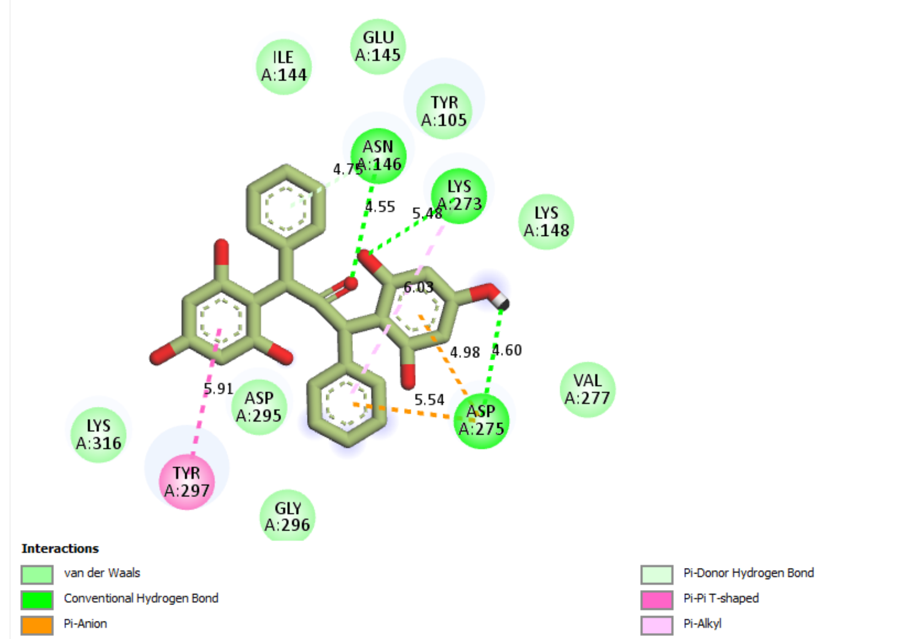
ILE A:144
GLU A:145
TYR A:105
ASN A:146
LYS A:273
LYS A:148
VAL A:277
ASP A:275
ASP A:295
LYS A:316
TYR A:297
GLY A:296
4.75
4.55
5.48
6.03
4.98
4.60
5.54
5.91
Interactions
van der Waals
Conventional Hydrogen Bond
Pi-Anion
Pi-Donor Hydrogen Bond
Pi-Pi T-shaped
Pi-Alkyl

<table>
<tr>
<td>Epigallocatechin 4-benzylthioether</td>
<td>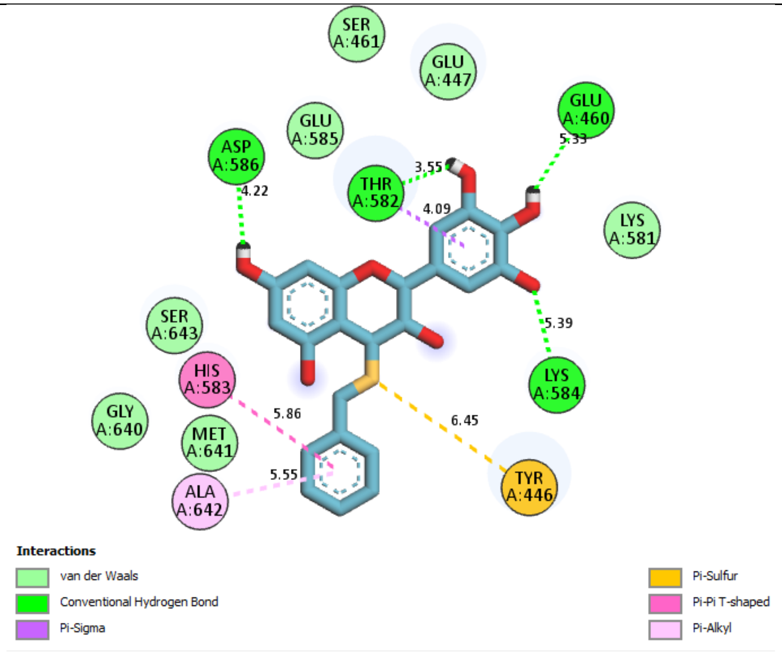
</td>
<td>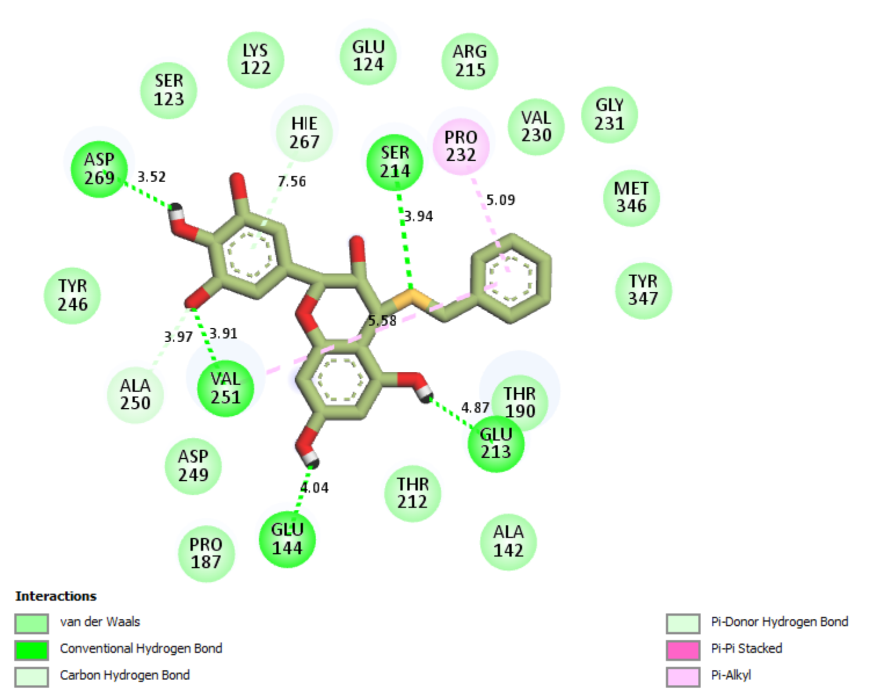
</td>
</tr>
</table>

<table>
<tr>
<td>Chroman-4-one</td>
<td>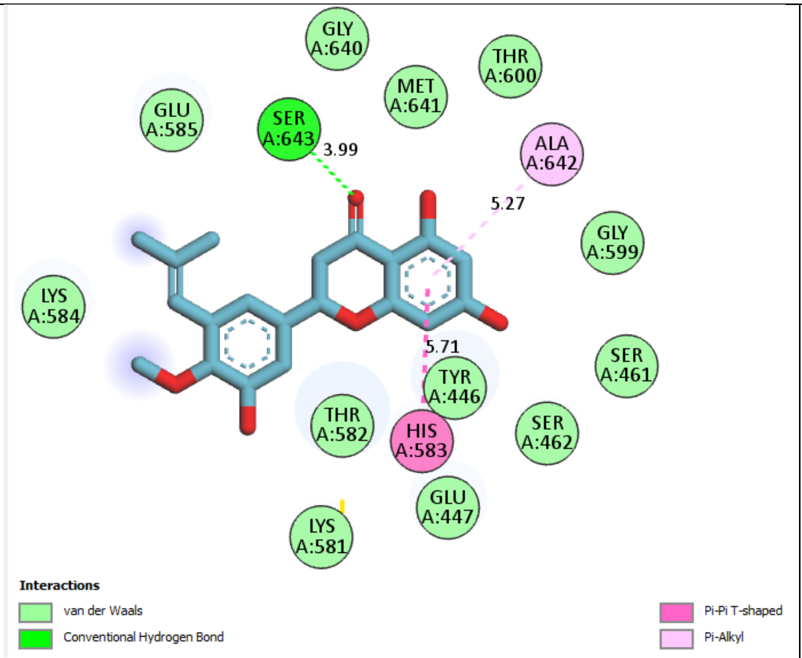
</td>
<td>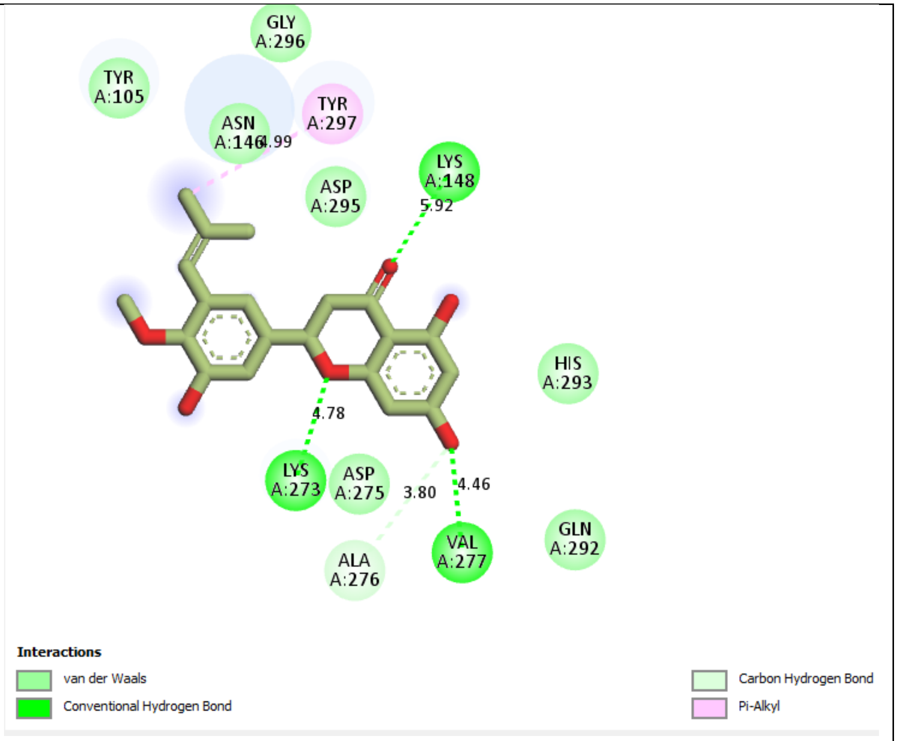
</td>
</tr>
</table>

Epicatechin gallate	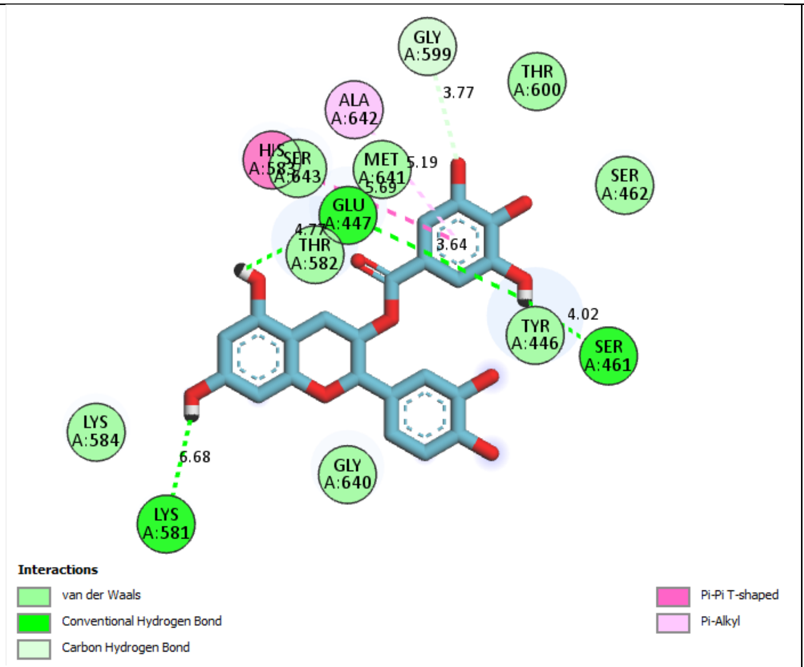	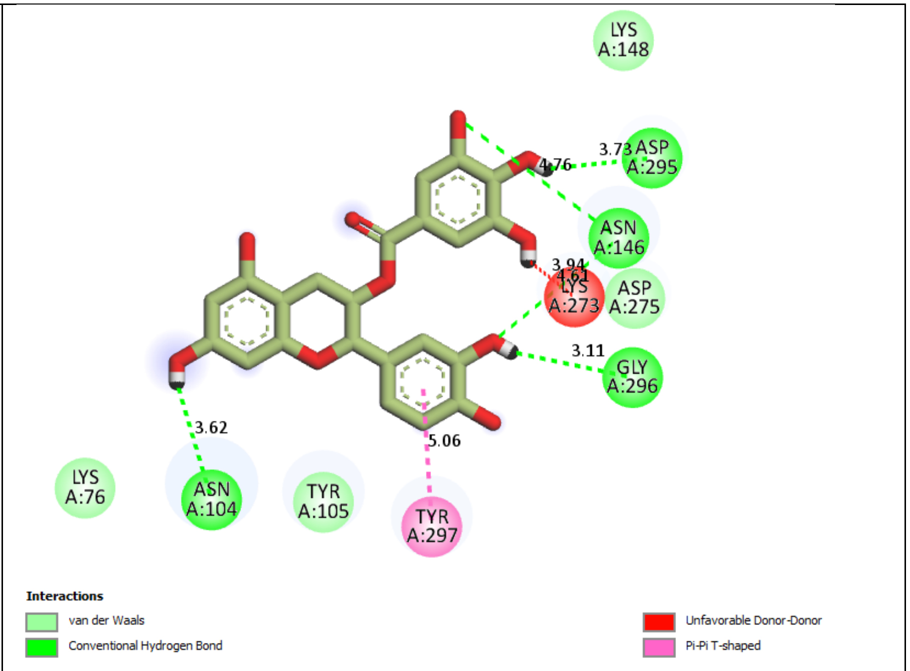
